# Associations between pain, health, and lifestyle factors in 10-year-old boys and girls from a Swedish birth cohort

**DOI:** 10.1186/s12887-023-04139-2

**Published:** 2023-06-29

**Authors:** Julia S. Malmborg, Josefine Roswall, Gerd Almquist-Tangen, Jovanna Dahlgren, Bernt Alm, Stefan Bergman

**Affiliations:** 1 School of Health and Welfare, Halmstad University, Box 823, SE-301 18 Halmstad, Sweden; 2 Spenshult Research and Development Centre, Bäckagårdsvägen 47, SE-302 74 Halmstad, Sweden; 3 Department of Pediatrics, The Sahlgrenska Academy, University of Gothenburg, SE-416 85 Gothenburg, Sweden; 4 Department of Pediatrics, Halland Hospital, SE-301 85 Halmstad, Sweden; 5 Child Health Care Unit, Region Halland, SE-301 80 Halmstad, Sweden; 6 Primary Health Care Unit, Department of Public Health and Community Medicine, Institute of Medicine, The Sahlgrenska Academy, University of Gothenburg, Box 454, SE-405 30 Gothenburg, Sweden

**Keywords:** Health-related quality of life, Sleep, Physical activity, Sedentary behavior, Pediatrics, Kidscreen-27

## Abstract

**Background:**

Pain is common in children and its associations with various biopsychosocial factors is complex. Comprehensive pain assessments could contribute to a better understanding of pediatric pain, but these assessments are scarce in literature. The aim of this study was to examine differences in pain prevalence and pain patterns in 10-year-old boys and girls from a Swedish birth cohort and to study associations between pain, health-related quality of life and various lifestyle factors stratified by sex.

**Methods:**

866 children (426 boys and 440 girls) and their parents from the “Halland Health and Growth Study” participated in this cross-sectional study. Children were categorized into two pain groups, “infrequent pain” (never–monthly pain) or “frequent pain” (weekly–almost daily pain), based on a pain mannequin. Univariate logistic regression analyses, stratified by sex, were performed to study associations between frequent pain and children’s self-reports of disease and disability and health-related quality of life (Kidscreen-27, five domains), and parents’ reports of their child’s sleep (quality and duration), physical activity time, sedentary time, and participation in organized physical activities.

**Results:**

The prevalence of frequent pain was 36.5% with no difference between boys and girls (*p* = 0.442). Boys with a longstanding disease or disability had higher odds of being in the frequent pain group (OR 2.167, 95% CI 1.168–4.020). Higher scores on health-related quality of life in all five domains for girls, and in two domains for boys, was associated with lower odds of being categorized into the frequent pain group. Frequent pain was associated with poor sleep quality (boys OR 2.533, 95% CI 1.243–5.162; girls OR 2.803, 95% CI 1.276–6.158) and more sedentary time (boys weekends OR 1.131, 95% CI 1.022–1.253; girls weekdays OR 1.137, 95% CI 1.032–1.253), but not with physical activity.

**Conclusions:**

The high prevalence of frequent pain needs to be acknowledged and treated by school health-care services and the healthcare sector in order to prevent pain from influencing health and lifestyle factors negatively in children.

## Background

Pediatric pain has been highlighted in a comprehensive report by The Lancet Child and Adolescent Health Commission [1], wherein it is described that pain may be underreported and undertreated in children and adolescents. Pain at a young age may recur or persist into adolescence [2] or adulthood [3], negatively influencing health and lifestyle factors [4, 5], which is why early attention and treatment of the problem is of great importance.

Pain is a biopsychosocial experience with multifaceted underlying factors [5–7], and is usually assessed in terms of localization, intensity, duration, and persistence [8, 9]. Reports of frequency, recall periods, and recurrence of pain vary greatly in the literature [8, 10, 11]. There is no gold standard for assessing pain, which makes both overall and region-specific pain prevalence difficult to determine in children and adolescents. It also makes it difficult to compare results between studies. Pain mannequins with a varying number of regions are frequently used to assess pain in children [6, 8, 12]. To enable analysis, the regions are usually grouped, but important aspects of pediatric pain characteristics, such as pain patterns, may be lost in the process. In the Lancet report [1], the authors request more comprehensive pain assessments, especially in birth cohorts, and that children should be followed throughout the various developmental stages and growth spurts.

In pain assessment, the biopsychosocial model is useful in trying to understand pain better. Several biopsychosocial factors have been identified as risk factors for pain. As regards biological factors, older age and female sex have been reported to be associated with pain in children and adolescents [10]. Both a too high [13, 14] or a too low [11] level of physical activity may be associated with pain. Poor sleep is another essential factor that may coexist with pediatric pain [15], as are lower scores on health-related quality of life assessments [16–18]. Children’s academic achievement [6, 19] may be an example of a social factor that interact with pain.

The Lancet report states that pain in children and adolescents needs to be acknowledged and assessed together with various biopsychosocial aspects over time [1]. In literature, pain and biopsychosocial factors are rarely stratified by sex in young children, meaning that potential effects of sex on pain and biopsychosocial factors may remain undiscovered. This study contributes by presenting pain measurements in 10-year-old children from an ongoing Swedish birth cohort research project. The aim of this study was to examine differences in pain prevalence and pain patterns in 10-year-old boys and girls from a Swedish birth cohort and to study associations between pain, health-related quality of life and various lifestyle factors stratified by sex.

## Methods

**Fig. 1:**
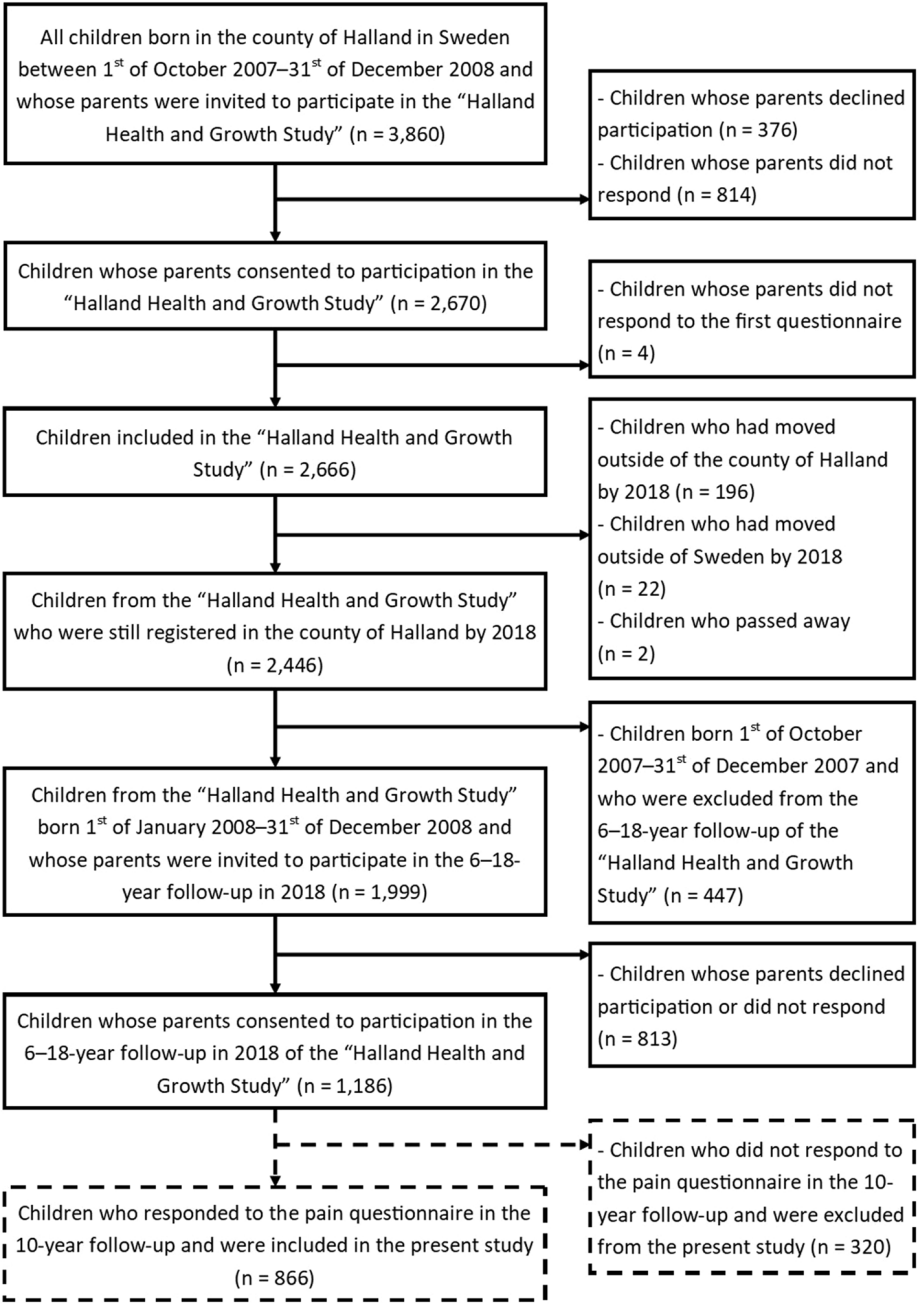
Flowchart with solid lines indicating the “Halland Health and Growth Study” and dashed lines indicating the final sample for the present study

All children born in the county of Halland in the south-west of Sweden between 1st of October 2007–31st of December 2008 and their parents were invited to participate in the “Halland Health and Growth Study” (H^2^GS) [20], a research project of a population-based birth cohort. In total, 3,860 children were born during the recruitment period and 2,666 children were recruited to the cohort. The first phase of H^2^GS covered ages 0–5 and in 2018 a request to continue participation in the second phase covering the ages 6–18 was carried out. In total, 1,186 children (45% of total baseline sample) were recruited to the 6–18-year follow-up. This cross-sectional study is part of the H^2^GS and includes a subgroup of 866 children that responded to a pain questionnaire at the 10-year follow-up (Fig. 1).

Questionnaires were distributed digitally at two occasions. In November 2018, children answered questions concerning longstanding disease or disability and health-related quality of life (HRQoL). In May 2019, children responded to a pain questionnaire, and parents answered questions about their child’s sleep (quality and duration), physical activity time, sedentary time, and participation in organized physical activities.

The research was carried out in accordance with the ethical guidelines presented in the Declaration of Helsinki [21]. The H^2^GS was approved by The Regional Ethical Review Board in Lund, Sweden (No. 299/2007) and The Swedish Ethical Review Authority (2018/141). Written informed consent was obtained from the parents. Participation was voluntary and participants could withdraw at any time without giving a reason. This study adhered to the Strengthening the Reporting of Observational studies in Epidemiology (STROBE) guidelines [22].

### Questionnaires

The presence of longstanding disease or disability was assessed by the question: “Do you have any longstanding disease or disability?” (yes / no). This was followed by the opportunity to report the type of longstanding disease or disability in free text.

Pain was evaluated by a pain mannequin [23]. The mannequin covered 20 body regions, including head and abdomen. Pain frequency (never / rarely / monthly / weekly / more than once a week / almost daily) was assessed for all regions [8]. Overall pain intensity during the last week was assessed by a numeric rating scale (NRS), ranging from 1 to 10, no pain–worst imaginable pain. Children were categorized into the pain groups of “infrequent pain” (never–monthly pain) or “frequent pain” (weekly–almost daily pain) from the highest reported pain frequency from at least one body region. Number of regions with frequent pain were also assessed and categorized into “1–2 regions” and “≥ 3 regions”.

HRQoL was measured by Kidscreen-27 [24]. The questionnaire covers five domains (“Physical well-being”, “Psychological well-being”, “Autonomy & parents”, “Peers & social support”, and “School environment”) in 27 questions. Answers were assessed according to the manual and results from each domain were transformed into T-values with a mean of 50 points and standard deviation of 10 points. Higher T-values indicate a better HRQoL [24].

Sleep was evaluated by quality and duration. Sleep quality was assessed by the question: “Does your child usually sleep well?” (yes / no). The answer “yes” indicated good sleep quality and the answer “no” indicated poor sleep quality. Sleep duration was assessed as average amount of sleep each night (reported in whole hours) and categorized into 6–8, 9 or 10–12 h.

Physical activity time and sedentary time was estimated as whole hours (1–10) on weekdays and weekends, respectively. Participation in organized physical activities during leisure time (yes / no) was also assessed.

### Statistical methods

Descriptive data with comparisons between boys and girls were analysed with the Student’s t-test and categorical data with the Chi^2^-test. Results were presented as mean values and standard deviations (SD) and percentage, respectively. Univariate logistic regression analyses were performed to study associations between the dependent variable (frequent pain) and the independent variables (disease or disability, pain intensity, HRQoL, sleep quality, sleep duration, physical activity time, sedentary time, and participation in organized physical activities). Results were presented as odds ratio (OR) with 95% confidence interval (CI) and stratified by sex. Statistical significance level was set at *p* < 0.05 and analyses were performed with IBM SPSS Statistics software v.28. (IBM Corp., Armonk, NY, USA).

## Results

Of the 1,186 children with parents who were recruited to the 6–18-year follow-up, 866 children (426 boys, 49.2% and 440 girls, 50.8%) answered the pain questionnaire at the 10-year follow-up and were, together with their parents, included in the present study.

### Pain prevalence and pain patterns

The prevalence of frequent pain was 36.5%, with no difference between boys and girls in pain group distribution (*p* = 0.442). Frequent pain from ≥ 3 regions was reported by 10.5% of the children (Table 1).

**Table 1:** Pain prevalence presented as n (%) and differences between boys and girls analysed with Chi^2^-test

	All, n (%) (*n* = 866)	Boys, n (%) (*n* = 426)	Girls, n (%) (*n* = 440)	*p*-value
Infrequent pain	550 (63.5%)	276 (64.8%)	274 (62.3%)	0.442^b^
Frequent pain^a^	316 (36.5%)	150 (35.2%)	166 (37.7%)	
1–2 regions	225 (26.0%)	109 (25.6%)	116 (26.4%)	
≥ 3 regions^c^	91 (10.5%)	41 (9.6%)	50 (11.4%)	0.404^d^

^a^Range 1–20 regions

^b^Frequent pain vs. infrequent pain

^c^3–13 regions for boys and 3–20 regions for girls reported

^d^Frequent pain in ≥ 3 regions vs. infrequent pain and frequent pain in 1–2 regions

Region-specific pain prevalence for frequent pain, irrespective of number of pain regions reported, is presented in Table 2. Girls reported a higher prevalence of head and abdominal pain than boys (*p* = 0.041 and *p* = 0.014, respectively), but no other differences in region-specific pain prevalence were found. Overall, the most commonly reported regions for frequent pain were head, abdomen and lower leg / foot.

**Table 2:** Region-specific pain prevalence for frequent pain presented as n (%) and differences between boys and girls analysed with Chi^2^-test by region

	All, n (%) (*n* = 866)	Boys, n (%) (*n* = 426)	Girls, n (%) (*n* = 440)	*p*-value
Anterior chest	23 (2.7%)	10 (2.3%)	13 (3.0%)	0.579
Neck	50 (5.8%)	23 (5.4%)	27 (6.1%)	0.642
Shoulder/upper arm left	19 (2.2%)	8 (1.9%)	11 (2.5%)	0.532
Shoulder/upper arm right	20 (2.3%)	9 (2.1%)	11 (2.5%)	0.704
Elbow/lower arm left	9 (1.0%)	6 (1.4%)	3 (0.7%)	0.334^a^
Elbow/lower arm right	12 (1.4%)	8 (1.9%)	4 (0.9%)	0.223
Upper back	13 (1.5%)	7 (1.6%)	6 (1.4%)	0.735
Low back	16 (1.8%)	9 (2.1%)	7 (1.6%)	0.569
Hand/wrist left	24 (2.8%)	12 (2.8%)	12 (2.7%)	0.936
Hand/wrist right	30 (3.5%)	14 (3.3%)	16 (3.6%)	0.778
Buttock left	2 (0.2%)	1 (0.2%)	1 (0.2%)	^b^
Buttock right	3 (0.3%)	2 (0.5%)	1 (0.2%)	^b^
Hip/upper leg left	18 (2.1%)	8 (1.9%)	10 (2.3%)	0.684
Hip/upper leg right	21 (2.4%)	11 (2.6%)	10 (2.3%)	0.767
Knee left	52 (6.0%)	25 (5.9%)	27 (6.1%)	0.868
Knee right	54 (6.2%)	22 (5.2%)	32 (7.3%)	0.200
Lower leg/foot left	81 (9.4%)	45 (10.6%)	36 (8.2%)	0.229
Lower leg/foot right	88 (10.2%)	47 (11.0%)	41 (9.3%)	0.404
Head	112 (12.9%)	45 (10.6%)	67 (15.2%)	0.041
Abdomen	103 (11.9%)	39 (9.2%)	64 (14.5%)	0.014

^a^Fisher’s exact test

^b^Results could not be analysed due to small numbers

Frequent pain was about twice as often reported to be bilateral than being unilateral, with bilateral pain in lower legs/feet displaying the highest prevalence (Table 3).

**Table 3:** Prevalence of frequent unilateral and bilateral pain by region and sex, presented as number (%)

	All, n (%) (*n* = 866)	Boys, n (%) (*n* = 426)	Girls, n (%) (*n* = 440)
Shoulders/upper arms			
Unilateral	7 (0.8%)	5 (1.2%)	2 (0.5%)
Bilateral	16 (1.8%)	6 (1.4%)	10 (2.3%)
Elbows/lower arms			
Unilateral	5 (0.6%)	4 (0.9%)	1 (0.2%)
Bilateral	8 (0.9%)	5 (1.2%)	3 (0.7%)
Hands/wrists			
Unilateral	12 (1.4%)	6 (1.4%)	6 (1.4%)
Bilateral	21 (2.4%)	10 (2.3%)	11 (2.5%)
Buttocks			
Unilateral	1 (0.1%)	1 (0.2%)	0 (0.0%)
Bilateral	2 (0.2%)	1 (0.2%)	1 (0.2%)
Hips/upper legs			
Unilateral	7 (0.8%)	5 (1.2%)	2 (0.5%)
Bilateral	16 (1.8%)	7 (1.6%)	9 (2.0%)
Knees			
Unilateral	28 (3.2%)	17 (4.0%)	11 (2.5%)
Bilateral	39 (4.5%)	15 (3.5%)	24 (5.5%)
Lower legs/feet			
Unilateral	31 (3.6%)	12 (2.8%)	19 (4.3%)
Bilateral	69 (8.0%)	40 (9.4%)	29 (6.6%)

### Descriptive data on health and lifestyle factors

In Kidscreen-27, girls reported higher values than boys in the domains of “Autonomy & parents”, “Peers & social support”, and “School environment”. A greater proportion of girls than boys participated in organized physical activities (Table 4).

**Table 4:** Descriptive data presented as n and mean ± standard deviation (SD) or as % and analysed with Students t-test or Chi^2^-test

		All (*n* = 866)		Boys (*n* = 426)		Girls (*n* = 440)		
		n	mean ± SD or %	n	mean ± SD or %	n	mean ± SD or %	*p*-value
Longstanding disease or disability	No	676	89.1%	320	87.2%	356	90.8%	0.110
	Yes	83	10.9%	47	12.8%	36	9.2%	
Pain intensity	NRS 1–10^a^	860	2.6 ± 1.7	425	2.5 ± 1.7	435	2.6 ± 1.8	0.193
Kidscreen-27^b^	Physical well-being	743	53.37 ± 8.81	358	53.41 ± 8.90	385	53.32 ± 8.73	0.892
	Psychological well-being	750	54.51 ± 9.47	360	54.33 ± 9.32	390	54.69 ± 9.61	0.604
	Autonomy & parents	727	56.44 ± 9.10	352	55.60 ± 8.77	375	57.23 ± 9.35	0.015
	Peers & social support	753	53.94 ± 9.03	364	53.23 ± 9.19	389	54.62 ± 8.83	0.034
	School environment	757	58.58 ± 8.78	364	57.65 ± 9.25	393	59.44 ± 8.24	0.005
Sleep quality	Good	734	92.2%	357	91.3%	377	93.1%	0.348
	Poor	62	7.8%	34	8.7%	28	6.9%	
Sleep duration	Range 6–12 h/n	782	9.2 ± 0.8	381	9.1 ± 0.8	401	9.2 ± 0.7	0.214
	6–8 h/n	113	14.5%	62	16.3%	51	12.7%	0.302
	9 h/n	411	52.6%	192	50.4%	219	54.6%	
	10–12 h/n	258	33.0%	127	33.3%	131	32.7%	
Physical activity weekdays	Range 1–10 h/d	797	2.9 ± 1.5	388	3.0 ± 1.4	409	2.9 ± 1.5	0.231
Physical activity weekends	Range 1–10 h/d	784	3.5 ± 1.8	380	3.6 ± 1.8	404	3.5 ± 1.8	0.147
Sedentary time weekdays	Range 1–10 h/d	789	5.3 ± 2.1	387	5.3 ± 2.2	402	5.2 ± 2.1	0.525
Sedentary time weekends	Range 1–10 h/d	772	4.8 ± 2.0	376	4.9 ± 2.1	396	4.7 ± 1.8	0.166
Participation in organized physical activities outside of school	Yes	705	88.0%	330	84.4%	375	91.5%	0.002
	No	96	12.0%	61	15.6%	35	8.5%	

*NRS* Numeric rating scale, *h/d* hours per day, *h/n* hours per night

^a^Scored from no pain–worst imaginable pain

^b^Scored from worst–best

### Associations between frequent pain and health and lifestyle factors

Boys with a longstanding disease or disability had higher odds of being in the frequent pain group (OR 2.167, 95% CI 1.168–4.020). Reporting a higher pain intensity during the last week was associated with frequent pain in both boys (OR 2.197, 95% CI 1.850–2.607) and girls (OR 2.398, 95% CI 2.008–2.864). Regarding HRQoL, higher scores in the domains of “Physical well-being” (OR 0.945, 95% CI 0.921–0.970), “Psychological well-being” (OR 0.953, 95% CI 0.931–0.975), “Autonomy & parents” (OR 0.957, 95% CI 0.934–0.980), “Peers & social support” (OR 0.958, 95% CI 0.935–0.981), and “School environment” (OR 0.952, 95% CI 0.928–0.977) for girls, and “Autonomy & parents” (OR 0.974, 95% CI 0.949–1.000) and “School environment” (OR 0.969, 95% CI 0.947–0.992) for boys, were associated with less risk of being categorized into the frequent pain group. Boys and girls with poor sleep quality were more likely to be categorized as having frequent pain (boys OR 2.533, 95% CI 1.243–5.162; girls OR 2.803, 95% CI 1.276–6.158), but the estimated sleep duration was not significantly associated with pain group categorization. Increases in the estimated amount of sedentary time was associated with having frequent pain for boys (weekends OR 1.131, 95% CI 1.022–1.253) and for girls (weekdays OR 1.137, 95% CI 1.032–1.253). No associations were found for physical activity (Table 5).

**Table 5:** Associations between frequent pain and health and lifestyle factors analysed with univariate logistic regressions, stratified by sex, and presented as odds ratio (OR) and 95% confidence intervals (CI)

		Boys			Girls		
		Frequent pain (*n* = 150) vs. Infrequent pain (*n* = 276)			Frequent pain (*n* = 166) vs. Infrequent pain (*n* = 274)		
		OR	95% CI	*p*-value	OR	95% CI	*p*-value
Longstanding disease or disability	No (ref)						
	Yes	2.167	1.168–4.020	0.014	1.943	0.976–3.870	0.059
Pain intensity	NRS 1–10^a^	2.197	1.850–2.607	< 0.001	2.398	2.008–2.864	< 0.001
Kidscreen-27^b^	Physical well-being	0.981	0.957–1.006	0.137	0.945	0.921–0.970	< 0.001
	Psychological well-being	0.978	0.955–1.001	0.063	0.953	0.931–0.975	< 0.001
	Autonomy & parents	0.974	0.949–1.000	0.047	0.957	0.934–0.980	< 0.001
	Peers & social support	0.982	0.959–1.005	0.120	0.958	0.935–0.981	< 0.001
	School environment	0.969	0.947–0.992	0.010	0.952	0.928–0.977	< 0.001
Sleep quality	Good (ref)						
	Poor	2.533	1.243–5.162	0.010	2.803	1.276–6.158	0.010
Sleep duration	9 h/n (ref)						
	10–12 h/n	0.853	0.534–1.364	0.507	0.809	0.515–1.271	0.358
	6–8 h/n	0.983	0.544–1.777	0.955	1.173	0.633–2.174	0.612
Physical activity weekdays	Range 1–10 h/d	0.994	0.860–1.148	0.930	1.085	0.953–1.236	0.217
Physical activity weekends	Range 1–10 h/d	0.955	0.848–1.075	0.447	1.011	0.904–1.131	0.850
Sedentary time weekdays	Range 1–10 h/d	1.100	0.998–1.212	0.055	1.137	1.032–1.253	0.009
Sedentary time weekends	Range 1–10 h/d	1.131	1.022–1.253	0.017	1.084	0.969–1.211	0.158
Participation in organized physical activities outside of school	Yes (ref)						
	No	1.213	0.692–2.126	0.501	0.992	0.484–2.031	0.982

*Ref* reference category, *NRS* numeric rating scale, *h/d* hours per day, *h/n* hours per night

^a^Scored from no pain–worst imaginable pain

^b^Scored from worst–best

## Discussion

The present study found that 36.5% of 10-year-olds reported having frequent pain in at least one body region. Furthermore, 10.5% reported having frequent pain in three or more body regions. Despite the fact that overall pain prevalence did not differ between boys and girls, analyses of the associations between frequent pain and health and lifestyle factors revealed both similarities and variations between the sexes. Reporting a higher HRQoL in all domains of Kidscreen-27 was associated with less risk of being categorized into the frequent pain group for girls, but only in some domains for boys. Higher pain intensity, poor sleep quality, and sedentary behavior were factors associated with higher risk of being categorized into the frequent pain group for all.

In the literature, there is great variety in reported pain prevalence for methodological reasons such as frequency, recall periods, regions, and recurrence of pain. The overall prevalence found in the current study is in line with previously reported findings of frequent pain in children, which ranges between 23 and 45% [17, 25, 26]. Overall pain prevalence did not differ between boys and girls in the current study. Pain is usually reported to be more common in girls [8, 17], but the prevalence gap between boys and girls seems to increase as children become adolescents and young adults [27]. Headache and abdominal pain were more frequently found in girls than boys in the present study, which is a finding that is also supported in previous research [5, 28, 29]. Regarding region-specific pain prevalence, it was surprising that the lower back was not among the most commonly reported pain regions, given that back pain generally is common in children and adolescents [8, 30].

In our current study, the reports that symmetrical, bilateral frequent pain was more common than unilateral frequent pain is an interesting finding, however, the groups were too small to perform statistical tests. Bilateral pain is often described as one of the main features of growing pains [31]. The etiology of growing pains is unknown [31], but many parents report that children have episodes of growing pains during childhood [32]. In the Lancet report [1], the authors request more comprehensive pain assessments throughout childhood. Symmetrical bilateral pain is not commonly reported in cohort studies, and following the development of bilateral pain over time might further add to our knowledge of pediatric pain.

Regarding factors associated with frequent pain in boys and girls in this study, interesting similarities and differences were found. Reporting a better score in all domains of HRQoL, as measured by Kidscreen-27, was associated with lower risk of being categorized into the frequent pain group for girls, but only in two domains for boys. In general, children and adolescents with pain usually score worse on HRQoL than those without pain [18, 33]. The same can also be seen in adolescent athletes [16]. The influence of pain on HRQoL seems to be more extensive in girls than in boys at 10 years of age. This could indicate that girls at an earlier age than boys show stronger associations between pain and low HRQoL, a phenomenon well known in adults with longstanding pain [34]. Interestingly, the relationship between having a longstanding disease or disability and frequent pain was more pronounced in boys than girls. More quantitative and qualitative research is needed in the area of health perception and pain in boys and girls.

Boys and girls whose parents reported that their child often displayed poor sleep quality were more likely to be categorized as having frequent pain. Compared to the 1980s, children and adolescents in Sweden today experience more sleeping problems [35]. Sleeping problems and pain coexist in children and adolescents [36], and in adults there seems to be a reciprocal relationship between sleep and pain [37]. Interestingly, sleep duration did not seem to influence pain group categorization. Associations between sleep and pain in children of the age group studied in the current study need to be explored in further research.

Physical activity level did not seem to influence pain group categorization in this age group in the present study. In adolescents, sports and a high level of physical activity have been identified as risk factors for pain [8, 11, 13, 14]. However, sedentary behavior was associated with being in the frequent pain group for both boys and girls. This finding is also supported in studies investigating a younger population [38] and in adolescents [9]. More studies of level of physical activity and sedentary behavior patterns in relation to pain are needed.

There are limitations that need to be discussed. A cross-sectional design does not allow for a determination of cause and effect of reported associations. There is also a time difference in the assessment of data that could affect the results, but due to the longstanding nature of the studied variables, the analyses have been treated as cross-sectional. This study represents the first pain measurements in the current birth cohort, and future follow-up measurements will enable further studies of associations. There may be disagreement between parental reports and children’s objectively measured physical activity and sedentary behavior [39]. The results from these items should be interpreted with caution. Both external dropout and internal dropout may affect the generalizability of the results. The internal dropout may be linked to the different time points at which the questionnaires were distributed, and this should be considered in future data collections. In larger longitudinal research projects, such as the H^2^GS, it is a careful trade-off between retrieving data and keeping participants motivated. Another potential limitation is that parental factors are not included in this study. These background factors are complex to interpret, and available parental factors did not show significant associations with children’s pain in preceding analyses. It was thus decided to focus this study on factors related directly towards the children.

The strengths of the study are the whole-body assessment of pain and the region-specific pain prevalence, which is not often presented for 10-year-old children. Another strength is that results are stratified by sex. This was particularly valuable because it allowed us to discern differences between boys and girls regarding health and lifestyle factors and their associations with frequent pain.

The high prevalence of frequent pain in this population-based study on school-aged children implicate that there is a need to proactively identify those at risk. The school health-care services and the healthcare sector could assist in this by increasing awareness and actively assessing pain in routine care.

## Conclusions

Frequent pain is common in children as young as 10 years of age, and one in ten experiences frequent pain in three or more body regions. In our current study, frequent pain was associated with poor sleep quality and sedentary time in both boys and girls. Reporting a better HRQoL was associated with lower likelihood of being categorized into the frequent pain group for girls, but only to some extent for boys. The high prevalence of frequent pain needs to be acknowledged and treated by school health-care services and the healthcare sector in order to prevent pain from influencing health and lifestyle factors negatively in children.

## Data Availability

The datasets generated during and/or analysed during the current study are not publicly available for ethical reasons and in line with Swedish legislation. Requests to make data available to reproduce the findings in the study should be made to the board of the Halland Health and Growth Study, represented by Maria V Andersson (maria.v.andersson@regionhalland.se) and Josefine Roswall (josefine.roswall@regionhalland.se).

## Abbreviations

CI: Confidence interval
HRQoL: Health-related quality of life
H/d: Hours per day
H/n: Hours per night
H^2^GS: Halland Health and Growth Study
NRS: Numeric rating scale
OR: Odds ratio
Ref: Reference category
SD: Standard deviation
STROBE: Strengthening the Reporting of Observational studies in Epidemiology

## References

[1] Eccleston C, Fisher E, Howard RF, Slater R, Forgeron P, Palermo TM, Birnie KA, Anderson BJ, Chambers CT, Crombez G, et al. Delivering transformative action in paediatric pain: a Lancet Child and Adolescent Health Commission. Lancet Child Adolesc Health. 2021;5(1):47–87. 10.1016/S2352-4642(20)30277-7 33064998

[2] Mikkelsson M, El-Metwally A, Kautiainen H, Auvinen A, Macfarlane GJ, Salminen JJ (2008). Onset, prognosis and risk factors for widespread pain in schoolchildren: a prospective 4-year follow-up study. Pain.

[3] Brattberg G (2004). Do pain problems in young school children persist into early adulthood? A 13-year follow-up. Eur J Pain.

[4] Eckhoff C, Straume B, Kvernmo S (2017). Multisite musculoskeletal pain in adolescence as a predictor of medical and social welfare benefits in young adulthood: the Norwegian Arctic Adolescent Health Cohort Study. Eur J Pain.

[5] Pourbordbari N, Riis A, Jensen MB, Olesen JL, Rathleff MS (2019). Poor prognosis of child and adolescent musculoskeletal pain: a systematic literature review. BMJ Open.

[6] Pourbordbari N, Jensen MB, Olesen JL, Holden S, Rathleff MS (2022). Bio-psycho-social characteristics and impact of musculoskeletal pain in one hundred children and adolescents consulting general practice. BMC Prim Care.

[7] Raja SN, Carr DB, Cohen M, Finnerup NB, Flor H, Gibson S, Keefe FJ, Mogil JS, Ringkamp M, Sluka KA, et al. The revised International Association for the Study of Pain definition of pain: concepts, challenges, and compromises. Pain. 2020;161(9):1976–82. 10.1097/j.pain.0000000000001939 PMC7680716 32694387

[8] Rathleff MS, Roos EM, Olesen JL, Rasmussen S (2013). High prevalence of daily and multi-site pain–a cross-sectional population-based study among 3000 danish adolescents. BMC Pediatr.

[9] Paananen MV, Auvinen JP, Taimela SP, Tammelin TH, Kantomaa MT, Ebeling HE, Taanila AM, Zitting PJ, Karppinen JI (2010). Psychosocial, mechanical, and metabolic factors in adolescents’ musculoskeletal pain in multiple locations: a cross-sectional study. Eur J Pain.

[10] King S, Chambers CT, Huguet A, MacNevin RC, McGrath PJ, Parker L, MacDonald AJ (2011). The epidemiology of chronic pain in children and adolescents revisited: a systematic review. Pain.

[11] Paananen MV, Taimela SP, Auvinen JP, Tammelin TH, Kantomaa MT, Ebeling HE, Taanila AM, Zitting PJ, Karppinen JI (2010). Risk factors for persistence of multiple musculoskeletal pains in adolescence: a 2-year follow-up study. Eur J Pain.

[12] Paananen M, Taimela S, Auvinen J, Tammelin T, Zitting P, Karppinen J (2011). Impact of self-reported musculoskeletal pain on health-related quality of life among young adults. Pain Med.

[13] Malmborg JS, Bremander A, Bergman S, Haglund E, Olsson MC (2022). Musculoskeletal pain and its association with health status, maturity, and sports performance in adolescent sport school students: a 2-year follow-up. BMC Sports Sci Med Rehabil.

[14] Kamada M, Abe T, Kitayuguchi J, Imamura F, Lee IM, Kadowaki M, Sawada SS, Miyachi M, Matsui Y, Uchio Y (2016). Dose-response relationship between sports activity and musculoskeletal pain in adolescents. Pain.

[15] Andreucci A, Campbell P, Richardson E, Chen Y, Dunn KM (2020). Sleep problems and psychological symptoms as predictors of musculoskeletal conditions in children and adolescents. Eur J Pain.

[16] Malmborg JS, Olsson MC, Bergman S, Bremander A (2018). Musculoskeletal pain and its association with maturity and sports performance in 14-year-old sport school students. BMJ Open Sport Exerc Med.

[17] Haraldstad K, Christophersen KA, Helseth S (2017). Health-related quality of life and pain in children and adolescents: a school survey. BMC Pediatr.

[18] Holden S, Rathleff MS, Roos EM, Jensen MB, Pourbordbari N, Graven-Nielsen T (2018). Pain patterns during adolescence can be grouped into four pain classes with distinct profiles: a study on a population based cohort of 2953 adolescents. Eur J Pain.

[19] Ragnarsson S, Johansson K, Bergstrom E, Sjoberg G, Hurtig AK, Petersen S. Perceived problems with academic achievement in school-aged children with recurrent pain - a longitudinal study. Scand J Public Health. 2021;49(5):487–94. 10.1177/1403494819889260 31826713

[20] Gerd AT, Bergman S, Dahlgren J, Roswall J, Alm B (2012). Factors associated with discontinuation of breastfeeding before 1 month of age. Acta Paediatr.

[21] General Assembly of the World Medical Association (2014). World Medical Association Declaration of Helsinki: ethical principles for medical research involving human subjects. J Am Coll Dent.

[22] von Elm E, Altman DG, Egger M, Pocock SJ, Gøtzsche PC, Vandenbroucke JP (2007). The strengthening the reporting of Observational Studies in Epidemiology (STROBE) statement: guidelines for reporting observational studies. Lancet.

[23] Bergman S, Herrstrom P, Hogstrom K, Petersson IF, Svensson B, Jacobsson LT (2001). Chronic musculoskeletal pain, prevalence rates, and sociodemographic associations in a swedish population study. J Rhuematol.

[24] The KIDSCREEN Group Europe (2006). The KIDSCREEN Questionnaires—Quality of life questionnaires for children and adolescents—handbook.

[25] Mikkelsson M, Salminen JJ, Kautiainen H (1997). Non-specific musculoskeletal pain in preadolescents. Prevalence and 1-year persistence. Pain.

[26] Fuglkjær S, Vach W, Hartvigsen J, Dissing KB, Junge T, Hestbæk L (2020). Musculoskeletal pain distribution in 1,000 danish schoolchildren aged 8–16 years. Chiropr Man Ther.

[27] Picavet HSJ, Gehring U, van Haselen A, Koppelman GH, van de Putte EM, Vader S, van der Wouden JHC, Schmits RJH, Smit HA, Wijga A (2021). A widening gap between boys and girls in musculoskeletal complaints, while growing up from age 11 to age 20 - the PIAMA birth cohort study. Eur J Pain.

[28] Wijga AH, Gehring U, van de Putte EM, Koppelman GH, Vader S, Schmits RJH, van der Wouden JC, Picavet HSJ (2021). Headache in girls and boys growing up from age 11 to 20 years: the Prevention and Incidence of Asthma and Mite Allergy birth cohort study. Pain.

[29] Holstein BE, Damsgaard MT, Ammitzbøll J, Madsen KR, Pedersen TP, Rasmussen M (2021). Recurrent abdominal pain among adolescents: trends and social inequality 1991–2018. Scand J Pain.

[30] Dissing KB, Hestbaek L, Hartvigsen J, Williams C, Kamper S, Boyle E, Wedderkopp N (2017). Spinal pain in danish school children - how often and how long? The CHAMPS Study-DK. BMC Musculoskelet Disord.

[31] Pavone V, Vescio A, Valenti F, Sapienza M, Sessa G, Testa G (2019). Growing pains: what do we know about etiology? A systematic review. World J Orthop.

[32] Lehman PJ, Carl RL (2017). Growing pains. Sports Health.

[33] Malmborg JS, Bremander A, Olsson MC, Bergman AC, Brorsson AS, Bergman S (2019). Worse health status, sleeping problems, and anxiety in 16-year-old students are associated with chronic musculoskeletal pain at three-year follow-up. BMC Public Health.

[34] Bergman S, Jacobsson LT, Herrström P, Petersson IF (2004). Health status as measured by SF-36 reflects changes and predicts outcome in chronic musculoskeletal pain: a 3-year follow up study in the general population. Pain.

[35] Norell-Clarke A, Hagquist C (2017). Changes in sleep habits between 1985 and 2013 among children and adolescents in Sweden. Scand J Public Health.

[36] Valrie CR, Bromberg MH, Palermo T, Schanberg LE (2013). A systematic review of sleep in pediatric pain populations. J Dev Behav Pediatr.

[37] Aili K, Nyman T, Svartengren M, Hillert L (2015). Sleep as a predictive factor for the onset and resolution of multi-site pain: a 5-year prospective study. Eur J Pain.

[38] Vierola A, Suominen AL, Lindi V, Viitasalo A, Ikävalko T, Lintu N, Väistö J, Kellokoski J, Närhi M, Lakka TA (2016). Associations of Sedentary Behavior, Physical Activity, Cardiorespiratory Fitness, and body Fat Content with Pain Conditions in children: the physical activity and Nutrition in Children Study. J Pain.

[39] Sarker H, Anderson LN, Borkhoff CM, Abreo K, Tremblay MS, Lebovic G, Maguire JL, Parkin PC, Birken CS (2015). Validation of parent-reported physical activity and sedentary time by accelerometry in young children. BMC Res Notes.

